# Does automated feedback impact the acceptability of AI-generated police body worn camera review? An implementation science natural experiment

**DOI:** 10.1007/s11292-025-09711-7

**Published:** 2025-11-24

**Authors:** Seth Watts, Brandon del Pozo, Michael D. White, Aili Malm

**Affiliations:** 1 School of Criminal Justice and Criminology, Texas State University, San Marcos, TX, USA; 2 The Warren Alpert Medical School of Brown University Health, Providence, RI, USA; 3 School of Criminology and Criminal Justice, Arizona State University, Phoenix, AZ, USA; 4 School of Criminal Justice and Emergency Management, California State University, Long Beach, Long Beach, CA, USA

## Abstract

The diffusion of innovations in policing has often been hindered by barriers to implementation and officer acceptance, which can derail an innovation regardless of its validity or effectiveness. Implementation Science (IS) is a useful lens for addressing such concerns because IS empirically examines the way a new technology or strategy is deployed, and it offers insights on barriers, facilitators, and fidelity. The present study uses an IS framework to investigate one of the latest innovations in policing: Artificial Intelligence (AI). In early 2024, two Arizona police departments deployed Truleo, an AI-driven body-worn camera review platform, via randomized controlled trials, but only one of those departments used a feature of Truleo that sends automated positive feedback emails to officers when they engage in behaviors assessed as “highly professional” by the AI algorithm. Using cross-sectional survey data from line-level officers (*n* = 84), we estimate both intent-to-treat and instrumental variable regression models to examine the effect of the automated emails on three implementation outcomes: acceptability, appropriateness, and feasibility. Officers in the department with automated feedback enabled reported higher levels of the appropriateness of Truleo. The instrumental variable models suggest that the automated emails resulted in higher levels of acceptability and appropriateness. The findings highlight: (1) the importance of evaluating different implementation strategies when deploying new technologies like AI, and (2) the potential value of providing AI-generated positive feedback to officers in the field as a means of ensuring the successful implementation of AI-driven officer accountability platforms in an agency.

## Introduction

The diffusion of innovations in policing is often met with resistance, particularly among the rank-and-file officers. A range of innovations, such as change in use of force policy (Terrill & Paoline, 2013), crisis intervention training (Bonfine et al., 2014), opioid use deflection programs (Formica et al., 2018), and body-worn cameras (Gaub et al., 2016), have been met with some form of resistance among line-level officers. Moreover, external actors who are tasked with implementing a new practice are frequently not trusted by rank-and-file officers (del Pozo et al., 2024c; Skogan, 2008) leading to a lack of acceptance or buy-in. The lack of acceptance among rank-and-file officers directly influences the success and sustainability of these programs, regardless of how effective they may be at achieving the ends that agency leadership intended for them. In plain terms, how can we expect an innovation to be effective if it is implemented poorly or is not accepted by the users?

Implementation science (IS) is a burgeoning field of research that specifically studies the barriers and facilitators of implementation, doing so by operationalizing variables that can empirically characterize the process of implementation as a set of measures separate from those that assess an innovation’s effectiveness (Bauer et al., 2015). For any given organizational change, both experiments and retrospective reviews can quantify these variables to understand what factors influenced a widely-accepted taxonomy of implementation outcomes: acceptability, adoption, appropriateness, feasibility, fidelity, implementation cost, penetration, and sustainability (Proctor et al., 2011, 2023). It is important to note that while these outcomes can determine the success or failure of an innovation, they concern the quality of the implementation, *and not whether a given innovation has the internal validity to produce the results under ideal conditions*.

In the experimental practice of IS, researchers can vary the parameters of a specific implementation variable to isolate and measure its effect on one or more implementation outcomes, ideally doing so under conditions of random assignment. To our knowledge, such an IS experiment has yet to be conducted in policing, a field in which IS remains acutely underleveraged (del Pozo et al., 2024) despite the well-established challenges of encouraging police departments to embrace innovation (del Pozo, Nevill, Pozo et al., 2024a, b, c), and extensive use of IS by researchers in health care settings (Bauer et al., 2015; del Pozo, Belenko, Taxman et al., 2024).

The use of artificial intelligence (AI) is among the latest high-profile innovations in policing, particularly AI-driven body-worn camera (BWC) analytics. In agencies where BWCs are used to record audio and video of police officers’ work in the field, the cameras produce enormous amounts of data that cannot possibly be directly reviewed by police supervisors given the immense time and cost that would be involved in such manual reviews. As a result, studies suggest that only a small portion of the BWC footage recorded by officers – 5% or less – is ever reviewed internally by police departments or externally by outside stakeholders (e.g., prosecutors, media, community members) (Freemon et al., 2023; Police Executive Research Forum, 2023; Uchida et al., 2022; Wickelgren, 2024). Agencies’ inability to review 95% or more of the footage recorded on BWCs likely undermines their effectiveness as a tool for accountability, transparency, deterrence, and incentivizing desirable police behaviors (Patterson & White, 2021; White et al., 2025).

AI can overcome this BWC review problem. This innovation offers the unique prospect of auditing police work in a much more efficient manner, as 100% of BWC footage can be analyzed by the AI platform in near real-time. However, there are many unanswered questions surrounding officer acceptance of AI and its use in policing. As with any innovation, the success of AI-driven BWC analytic platforms depends not only on their inherent ability to perform as desired by agency leaders, but also on how successfully they are implemented as a form of organizational change, an outcome which can be heavily influenced by how such change is received by rank and file officers (del Pozo et al., 2024; del Pozo, Nevill, Pozo et al., 2024a, b, c). In the case of AI-driven BWC review, change has been met with resistance in several cases. For example, police unions in Seattle, Washington, and Vallejo, California pushed to stop the adoption of AI-BWC analytics (Carter, 2023; Sault, 2023), and despite findings that the use of Truleo was associated with overall improvements in police professionalism (Adams et al., 2024), the Aurora, Colorado Police Department let its contract expire with no plans to continue the platform’s use (Bradbury, 2024).

To provide a novel example of IS research in policing, this study utilized a natural experiment to test the effect of automated positive feedback via an AI-based platform (Truleo) on frontline users’ perceptions of three implementation-specific outcomes: the acceptance, appropriateness, and feasibility of AI-driven automated BWC review technology. In the study, two similarly situated Arizona police departments deployed Truleo via randomized controlled trials (RCTs), but one police department used the automated positive feedback feature of Truleo while the other did not. Importantly, the present study therefore focuses on *how* Truleo was deployed in two departments as a potential factor influencing the platform’s implementation-specific outcomes. Specifically, audit and feedback has been cited in the healthcare field as an implementation strategy (Powell et al., 2015), and this study assessed if an AI-driven audit of behavior followed by automated positive feedback influenced officer attitudes about the use of AI in reviewing the BWC footage they generate. The findings suggest that automated feedback positively influences officers’ perceptions of acceptability and appropriateness. This has implications for both the general practice of IS research in policing, as well as for police practitioners determining the most effective ways to successfully implement AI-driven platforms in their agencies.

### Literature review

#### Innovations in policing

Innovation in policing has taken many different forms over the last century (Kelling & Moore, 1988). One of the most notable shifts began in the 1980s and 1990s when the focus on innovation in policing prioritized reforms and technologies based on empirically validated effects, most notably, crime reduction (Braga & Weisburd, 2006; Sherman, 1998). This marked the beginning of the Evidence-Based Policing (EBP) movement, which sought to direct finite resources to policies, strategies, and technologies that had been scientifically evaluated (Sherman, 1998). Over the last 40 years, countless innovations have been evaluated and implemented in police departments across the globe including crime-control strategies (e.g., hot spots policing, problem-oriented policing, focused deterrence), technological innovations (e.g., dashboard cameras, less than lethal impact devices, CompStat, BWCs), training (e.g., crisis intervention training, de-escalation, implicit bias training), among many others (e.g., use of force policy changes, pursuit policies, investigative techniques, naloxone programs).

Despite this steadily growing body of rigorous experimental and quasi-experimental research on innovation in policing, there remains resistance to adopting new reforms or technologies in police agencies. Though professional resistance to change can occur for a variety of reasons (Skogan, 2008), officer buy-in and acceptance has direct implications for the implementation of a given innovation. If front-line users do not buy in to the innovation, it could defeat efforts to effectively incorporate it into daily operations, achieve desired goals, and sustain the practice in the long term (del Pozo et al., 2024c; Formica et al., 2018; Lum et al., 2017).

### Implementation science

The IS framework is a systematic way for understanding how implementation can impact acceptance of an innovation. Broadly, IS evolved from medicine and health care’s realization approximately two decades ago that despite a commitment to evidence-based practice, the implementation of well-proven innovations could nonetheless take nearly 17 years on average (Morris et al., 2011), face substantial resistance from physicians and other practitioners (Greenhalgh et al., 2014; Pope, 2003), and flounder or fail because of poor implementation, even if the innovation itself had proven valid, efficacious, and effective in research settings (Bauer et al., 2015). As a field that arose directly from the movement toward evidence-based medicine (Sherman, 1998), evidence-based policing suffers from the same challenges, but has yet to develop the rigorous, often experimental study of implementation as an integral companion to assessing traditional measures of an innovation’s effectiveness (del Pozo et al., 2024). There has been very little IS research in police settings, and it remains an underutilized asset in accelerating police organizational change.

To jumpstart that process, researchers have developed an overview of IS for police researchers and practitioners (del Pozo et al., 2024), and collected pilot data about the determinants of successful implementation in policing (del Pozo, Nevill, Pozo et al., 2024a, b, c). Central to such efforts are a range of evolving frameworks and measures designed to operationalize and study implementation variables. Among them, the Consolidated Framework for Implementation Research (CFIR, 2023) has proven to be highly adaptable to a variety of settings (Kirk et al., 2016), as it consists of an inventory of dozens of variables both within and outside of an organization that have been shown to act as barriers and facilitators of organizational change. These variables can be used to identify what are thought to be the most appropriate implementation strategies, and the results of experimentation and measurement can be used to assess Proctor et al.’s (2011, 2023) widely accepted taxonomy of implementation outcomes. This study follows such an approach.

### BWCs and AI

AI is one of the more recent innovations that has made its way into policing. Though AI has numerous potential applications in policing (Europol, 2024), its utility for efficiently auditing BWC footage – and overcoming the 5% problem – is among the most promising (Watts et al., 2024; White et al., 2025). That is, approximately 5% of all BWC footage that is produced is ever reviewed – by anyone (Police Executive Research Forum, 2023; Uchida et al., 2022; Wickelgren, 2024). The 5% problem likely undermines many of the stated goals of BWCs (e.g., accountability and transparency). Moreover, BWCs are thought to generate a “civilizing effect” leading to reductions in use of force and complaints against officers (White, 2014), but this effect hinges on a belief the footage will be reviewed. The 5% problem likely contributes to a diminished capacity for any civilizing effect to take place, as the inability to review BWC footage means that officer and/or citizen behavior is not observed and thus there is no effective deterrent in place (Patterson & White, 2021). White et al. (2025) suggests this may contribute to the mixed effects of BWCs (Lum et al., 2020).

The main approach to overcoming the 5% problem is to devise a means of reviewing a substantial enough sample of an agency’s BWC footage to be confident in the agency’s ability to detect undesirable behaviors and provide effective behavioral incentives to police. However, it is not possible for administrators or data analysts to conduct such a review manually because of the sheer amount of BWC footage. For instance, Freemon et al. (2023) found that there was over 483,000 h of BWC footage uploaded in the Phoenix Police Department in 2020 alone. Additionally, the New York Police Department ingested approximately 130,000 BWC-recorded encounters per week in 2019 (Garnett, 2021: 12). Moreover, this is not just an issue for large police departments – it is a problem that affects small police agencies as well, as both the volume of BWC footage and the staff hours available for BWC review decrease in rough proportion to each other (Wickelgren, 2024).

A solution to this problem is what many of the AI-driven BWC analytics platforms have targeted as a selling point. Using AI algorithms for automated review, a growing number of commercial vendors provide platforms that can review 100% of the BWC footage that is produced. The most notable platforms that offer these services include Truleo, Axon, and Polis Solutions. These platforms provide the opportunity for police departments to utilize *all* their BWC footage data to monitor and improve officer behavior through a variety of interventions (e.g., positive reinforcement, training, coaching, etc.), with the prospect of enhancing police community relations in doing so and ultimately making a police agency more efficient and effective at its work.

Indeed, there is a growing interest in these platforms across the country and globally. However, there are concerns from both within departments and among community members about the acceptance of AI and how departments may use AI-based platforms (Adams, 2025; Carter, 2023; Porter & Ogdon, 2023). Focusing specifically on front-line users, buy-in is critical for the effectiveness of any technological innovation to be realized. Given this need, IS offers the opportunity to rigorously understand what factors may influence the ultimate acceptability, adoption, and feasibility of an AI-driven BWC review platform among rank-and-file officers.

### What we know about officer perceptions of AI in policing

Research on perceptions of AI within policing is limited. Of the studies that have examined perceptions of AI in the workplace, researchers have focused on BWC integration with AI (Watts et al., 2024; White et al., 2025), AI-generated feedback (Adams, 2025), and AI-assisted report writing (Boehme et al., 2025). Watts et al. (2024) surveyed officers about their perceptions of Truleo in the months prior to its deployment. The authors found that, in general, perceptions of Truleo were neutral, though upper-level management viewed the technology in a more positive light than line-level officers. In a follow-up study looking at changes in perceptions following deployment of the intervention, White et al. (2025) found that there were no changes in perceptions of Truleo other than improved knowledge of the platform and less concern for how it would be used by supervisors. Additionally, in weekly and monthly surveys of officers and sergeants using the platform, officers noted that over time Truleo provided areas to improve and that Truleo provided useful information. Sergeants noted that the Truleo platform demonstrated officers’ positive performance.

In an experimental survey, Adams (2025) found that officers viewed AI-generated feedback much more negatively than supervisor-mediated feedback, casting doubt on the potential positive influence of automated feedback if not suggesting it may be an impediment to the acceptability and adoption of an AI platform. Boehme et al. (2025) showed that while there were no significant differences in perceptions between users’ and non-users’ of an AI-assisted report writing software, there were some positive qualitative findings. Namely, supervisors reported higher quality reports from their officers. In the limited research to date, officer acceptance of AI is by no means guaranteed. This line of inquiry is particularly important, as line-level officers’ perceptions can shape the adoption and diffusion of innovative technologies both within and across agencies. Theoretically, officers who view AI positively owing to successful aspects of its implementation may be more likely to accept and act on information provided by AI, and to share positive experiences with their peer networks, leading to greater acceptance of the innovation among the police profession. Similarly, if Truleo, the focus of the present study, improves officers’ professionalism (see Adams et al., 2024), low acceptance could short-circuit any positive behavioral changes. This rationale sets the stage for applying IS to the study of this innovation in police practice.

## Methods

### Setting

The current study is part of a larger project evaluating the impact of Truleo in three Arizona law enforcement agencies (Arizona Department of Public Safety, Apache Junction Police Department (AJPD), and Casa Grande Police Department (CGPD). The larger project is focused on the adoption of Truleo via three RCTs in the respective police departments. The primary objectives of the larger study center on evaluating officers’ receptivity to and the causal impact of Truleo on officer behavior. For the current study, we focus on AJPD and CGPD, two mid-sized agencies that deployed Truleo via RCTs in early 2024. Both police departments have approximately the same number of sworn personnel (75–85), experience similar crime and violence problems, and are situated approximately 50 miles apart in the Phoenix metropolitan area. Apache Junction has a population of 41,153 with just over 12% living in poverty. The city is predominately White (82.5%) with 18% being Hispanic (United States Census Quick Facts, 2024a). Casa Grande has a population of approximately 63,743 with just under 15% living in poverty. The city is predominately White (64.7%) with a sizable Hispanic population (44.6%) (United States Census Quick Facts, 2024b).

For context, the RCTs in both police departments included patrol, traffic, and special unit officers. Thirty-five officers were randomized in AJPD, and 55 officers were randomized in CGPD. The RCTs were six months long with CGPD starting on February 1, 2024, and AJPD starting on March 22, 2024. Those in the Treatment group had access to their Truleo platform, as did their Sergeants. Control group officers did not have access to their Truleo platform and neither did their Sergeants.

### Truleo’s platform

Truleo offers police departments an AI-driven platform that audits the audio of BWC footage by producing a text-based transcript that is processed via a natural language processing algorithm, which then tags specific phrases or words with labels that may be of interest (e.g., impolite language, use of force, de-escalation). This process takes place following an officer’s shift once they have docked their BWC. According to Truleo, the transcribing and labeling process is over 90% accurate (Shastry, 2022). While this is the core feature of the platform, other features include report writing assistance and AI-assisted search engines focused on department policies and state statutes (see https://truleo.co/truassist).

One of the key outcomes that is produced by Truleo is the autogenerated Professionalism outcome. Each encounter recorded on BWC and analyzed by Truleo is classified on a tripartite Professionalism measure regardless of the department or officers’ level of access to the platform.^1^ Encounters are labeled “Highly Professional” if an officer (a) uses 25 or more words before taking an official action, (b) does not threaten the use of force, (c) does not use force, and (d) does not use impolite language (e.g., directed profanity). “Substandard Professionalism” includes encounters where an officer uses impolite language. Importantly, for an encounter to be labeled “Substandard” a supervisor must acknowledge that the officer did in fact use impolite language and that it was against department policy or expectations. This is considered the “human in the loop” aspect of the platform that attempts to minimize false positives and allows for contextual factors to be considered. “Standard Professionalism” is in-between these two categorizations and generally means that the encounter met department standards. ^2^

### The natural experiment

This implementation science study takes place in CFIR’s Inner Setting domain, the internal organizational environment where an innovation takes place, and where organizational culture and the readiness of rank-and-file employees for a given change can affect successful implementation (CFIR, 2023). Once a police organization begins using the product, Truleo’s platform provides the option of sending out automated emails to line-level officers when they have an encounter that is rated as “Highly Professional.” The automated positive feedback feature is a central component of the Truleo platform that is designed to formally recognize highly professional behavior by officers (e.g., positive reinforcement). Given that most good police work goes unnoticed (Fyfe, 1988), this feature brings highly professional policing to the attention of the officers and their chain of command. In plain terms, the platform overcomes a longstanding problem in policing by systematically capturing good policing in near real time, allowing line-level officers and their supervisors to note and review professional police work following each highly professional encounter.

The automated feedback feature was active for the duration of the six-month RCT in the CGPD (February 1, 2024 – July 31, 2024). In the AJPD six-month RCT (March 22, 2024 – September 21, 2024), the automated feedback feature was disabled. The decision to disable the automated feedback feature was made by the AJPD leadership in consultation with the research team. The reason for this is because the automated feedback mechanism within Truleo could only be disabled at the department level, meaning that both Treatment and Control officers in the RCT would get automated notifications of “Highly Professional” encounters. Given that this is a source of contamination for the RCT, the feature was disabled in AJPD to attempt to (a) limit this source of contamination and (b) attempt to capture the impact of the automated emails on officer acceptance.

The automated feedback feature was the sole observable difference in the implementation of the Truleo intervention in two similarly situated police departments. Randomization was carried out the same way. The officers involved were in the same assignments (patrol, traffic, specialty units), and the research design, survey and data collection protocols were identical across agencies. Access to and use of Truleo was also identical, except for the automated feedback feature. This difference allowed us to assess whether the automated, AI-generated positive feedback feature influenced key outcomes of interest in implementation research: *acceptability*, *appropriateness*, and *feasibility*, which are discussed below. Our research questions are:

1. Does automated positive feedback increase officers’ perceptions of Truleo’s acceptability?
2. Does automated positive feedback increase officers’ perceptions of Truleo’s appropriateness?
3. Does automated positive feedback increase officers’ perceptions of Truleo’s feasibility?

### Data collection

We administered a Qualtrics survey to officers in both police departments approximately five months into the RCT study period.^3^ Participation was voluntary, the instrument was anonymous and took approximately 10 min to complete. The survey was shared internally by each department’s leadership on our behalf with informed consent language included. The survey primarily consisted of 5-point Likert scale items (1 = Strongly disagree, 2 = Disagree, 3 = Neutral, 4 =Agree, 5 = Strongly agree). The survey items focused primarily on officers’ attitudes about Truleo, including their understanding of the platform, its utility, and concerns about its use. The post-intervention surveys were distributed on July 19, 2024 (CGPD) and August 16, 2024 (AJPD). The survey portal accepted responses for approximately 3 weeks following the distribution with a reminder email from leadership at one week. In AJPD, the response rate was 76%, and in CGPD, the response rate was 82%. For the current study, the first author’s University IRB approved the survey on November 1, 2023, and the full study on January 18, 2024 (STUDY00018951).

### Dependent variables

We focused on three established IS framework composite measures of *acceptability*, *appropriateness*, and *feasibility*. The measures used in the present study were adopted from Weiner et al. (2017), which affirmed the validity of their psychometric properties when administered in healthcare settings. As this study is the first IS experiment of its kind in policing to our knowledge, and is a demonstration project in that regard, we adapted these items as written under the assumption their validity would hold in a police setting. In that vein, we used two items from each construct with a high Cronbach’s alpha to develop the three composite measures (see Weiner et al., 2017; Table 1). To capture *acceptability*, we used the items “I approve of the use of Truleo to monitor performance” and “I think Truleo is a satisfactory product” (*α* = 0.926); to capture *appropriateness*, we used the items “Truleo is a good tool for assessing performance in the field” and “Truleo meets the needs it is designed to meet” (*α* = 0.932); and to capture *feasibility*, we used the items “Truleo is a viable way to provide feedback” and “Truleo is easy to use” (*α* = 0.823). All items were measured on a five-point Likert semantic scale with anchoring language (1 = Strongly disagree, 3 = Neutral, 5 = Strongly agree).

### Independent variable and covariates

As mentioned above, the natural experiment took place at the department level. Thus, following an intent-to-treat (ITT) approach, our primary independent variable of interest is the department identifier (CGPD or AJPD), effectively capturing assignment to receive automated emails from Truleo regarding users’ highly professional encounters (CGPD) or not (AJPD). Additionally, in our covariate adjusted models, we controlled for officer-level characteristics including assignment (patrol [ref = non-patrol]), RCT treatment assignment (Treatment [ref = Control]),^4^ gender (female [ref = male]), race (Black, Hispanic, Other [ref = White]), age, and education (Bachelor’s degree or higher [ref = less than a Bachelor’s degree]). The RCT treatment assignment variable (Treatment v. Control) deserves a bit more attention. Given the department-level implementation of the automated feedback feature of Truleo, we actually have four variations of the Truleo intervention across the two departments (see Table A1):

- AJPD Control: full control (no Truleo access, no automated feedback).
- AJPD Treatment: partial intervention (yes Truleo access, no automated feedback).
- CGPD Control: partial intervention (no Truleo access, yes automated feedback).
- CGPD Treatment: full intervention (yes Truleo access, yes automated feedback).

Additionally, to help control for other department-level and sergeant specific confounders, we included two other variables: *Organizational justice* and *perceived supervisor support*. Both measures include six items consisting of 5-point Likert scales (1 = Strongly disagree, 3 = Neutral, 5 = Strongly agree). We then standardized each measure with a mean of 0 and standard deviation of 1. *Organizational justice* includes the following items: “Top management will publicly recognize an officer’s good performance,” “Promotions reflect merit and contributions to the agency,” “When written up for a minor rule violation, officers are treated fairly by management,” “Department leadership is very interested in the personal welfare of their officers,” “The department’s discipline process is fair,” “The department’s promotional process is fair” (*α* = 0.89). *Perceived supervisory support* includes the following items: [My direct supervisor…] “Values my contribution to the agency’s success,” “Considers my best interests when it makes decisions that affect me,” “Values my opinions,” “Takes pride in my work accomplishments,” “Cares about my general satisfaction at work,” “Provides support when I have a problem” (*α* = 0.97).

### Analytical sample and plan

Given our research questions, we focused specifically on the perceptions of non-supervisory, line-level officers. Other respondents (e.g., sergeants, command staff) were excluded from our analytical sample. We employed ordered-logistic regression models with robust standard errors to handle the ordinal nature of our outcome variables (Brauer & Day, 2025) and heteroskedasticity. For each outcome we present unadjusted and adjusted models focusing on the department identifier coefficient. The equation is as follows:

$$P\left(Y_{i}\leq j\right)=\frac{1}{1+\exp\left[-\left(\mu_{j}-\beta_{1}T_{i}-X_{i}\gamma \right)\right]}\;\text{for}\;j=1,2,\ldots ,J-1$$

Where.

$Y_{i}$ = the ordinal outcome (acceptability, appropriateness, or feasibility).

$T_{i}$ = treatment assignment (CGPD = 1; automated emails turned on).

$X_{i}$ = a vector of covariates (organizational justice, perceived supervisor support, RCT treatment, patrol, gender, race, age, education) in the adjusted models.

$\mu_{j}$ = the cut points between the ordinal values of the outcome.

We then used an instrumental variable (IV) approach with the department identifier as the source of exogenous variation to estimate a local average treatment effect (LATE) among those who report receiving an email in CGPD. Importantly, this method helps reduce the noise around officers’ self-reporting of receiving an email. For instance, officers who are more professional likely have other correlated characteristics that might affect their perceptions of Truleo’s platform. Using the department identifier as a source of plausible exogenous variation to predict officers’ perceptions *through* the automated highly professional email narrows the focus to variation predicted by the department. We employed a Two Stage Least Squares (2SLS) approach for our instrumental variable models using robust standard errors. Although our outcome is nonlinear, Basu et al. (2018) suggest that the 2SLS is robust when dealing with nonlinear outcomes. Our first stage and second stage regression models are below:

First stage:

$$truleo\_email_{i}=\pi_{0}+\pi_{1}dept_{i}+\pi_{2}RCT\text{\_}treatment_{i}+\pi_{3}patrol_{i}+\;\;\pi_{4}age_{i}+\pi_{5}female_{i}+\pi_{6}college\_degree_{i}+\sum_{r}\delta_{r}race_{ri}+u_{i}$$

Where.

$truleo\_email_{i}$ = the endogenous variable correlated with the error term (e.g., reported receiving an email).

$dept_{i}$ = the instrument (e.g., department identifier [ref =AJPD]).

$\pi_{1}$ = the coefficient for the instrument variable.

$\pi_{2}-\pi_{6}$ = control variable coefficients for organizational justice, perceived supervisor support, RCT treatment, patrol, age, gender, education.

$\delta_{r}$ = the coefficients for the race dummy variables.

$u_{i}$ = the error term in the first stage.

Second stage:

$$Y_{i}=\beta_{0}+\beta_{1}trule\;\hat{eo\text{\_}email}i_{i}+\beta_{2}RCT\text{\_}treatment_{i}+\beta_{3}patrol_{i}+\;\beta_{4}age_{i}+\beta_{5}female_{i}+\beta_{6}college\_degree_{i}+\sum_{r}\gamma_{r}race_{ri}+\epsilon_{i}$$

Where.

$Y_{i}$ = the outcome of interest (acceptance, appropriateness, and feasibility).

$trule\;\hat{eo\text{\_}email}i_{i}$ = the predicted value of the endogenous regressor from the first stage.

$\beta_{1}$ = the coefficient of interest, capturing the causal effect of receiving an email on $Y_{i}$.

$\beta_{2}-\beta_{6}$ = the coefficients for control variables organizational justice, perceived supervisor support, RCT treatment, patrol, age, gender, education.

$\gamma_{r}$ = the coefficients for the race indicator dummy variables.

$\epsilon_{i}$ = the error term in the second stage.

We report sample summary statistics, ordered logistic regression, and IV regression models’ findings for our three primary outcomes (acceptance, appropriateness, and feasibility) below. We also include alternative specifications in the supplemental file including collapsing the 5-point Likert scale to a 3-point Likert scale and employing OLS regression models (Tables A9-A12). All analyses were conducted in Stata 17 (StataCorp, 2021).

## Results

### Sample characteristics

Our sample consists of 84 survey responses from frontline officers (81 officers, 3 corporals) between the two departments (AJPD = 43, CGPD = 41). Table 1 presents the descriptive statistics of the analytical sample. Respondents from the two departments were mostly assigned to patrol (58% and 73%), male (87% and 90%), aged between 36 and 39 – on average, predominately white (64% and 54%), and without a college degree (30% and 21% reported having a Bachelor’s degree or higher). Additionally, in AJPD and CGPD, 42% and 85%, respectively, report having received an email regarding a “highly professional” encounter.

Tables A2-A5 explore the two police departments’ demographics to ensure they are similar. We show that (a) their perceptions of highly correlated proxies for our outcomes were similar in the pre-intervention survey (Table A5),^5^ (b) their demographics did not differ significantly in the pre-period (Table A2), (c) their demographics did not differ significantly by department in the post-period (Table A3), and (d) their demographics did not differ significantly between pre- and post-periods (Table A4). These findings, along with the fact both departments were amid a RCT receiving the same intervention (except for the automated emails), provide support that the two departments and their officers are largely similar based on observable characteristics.

**Fig. 1:**
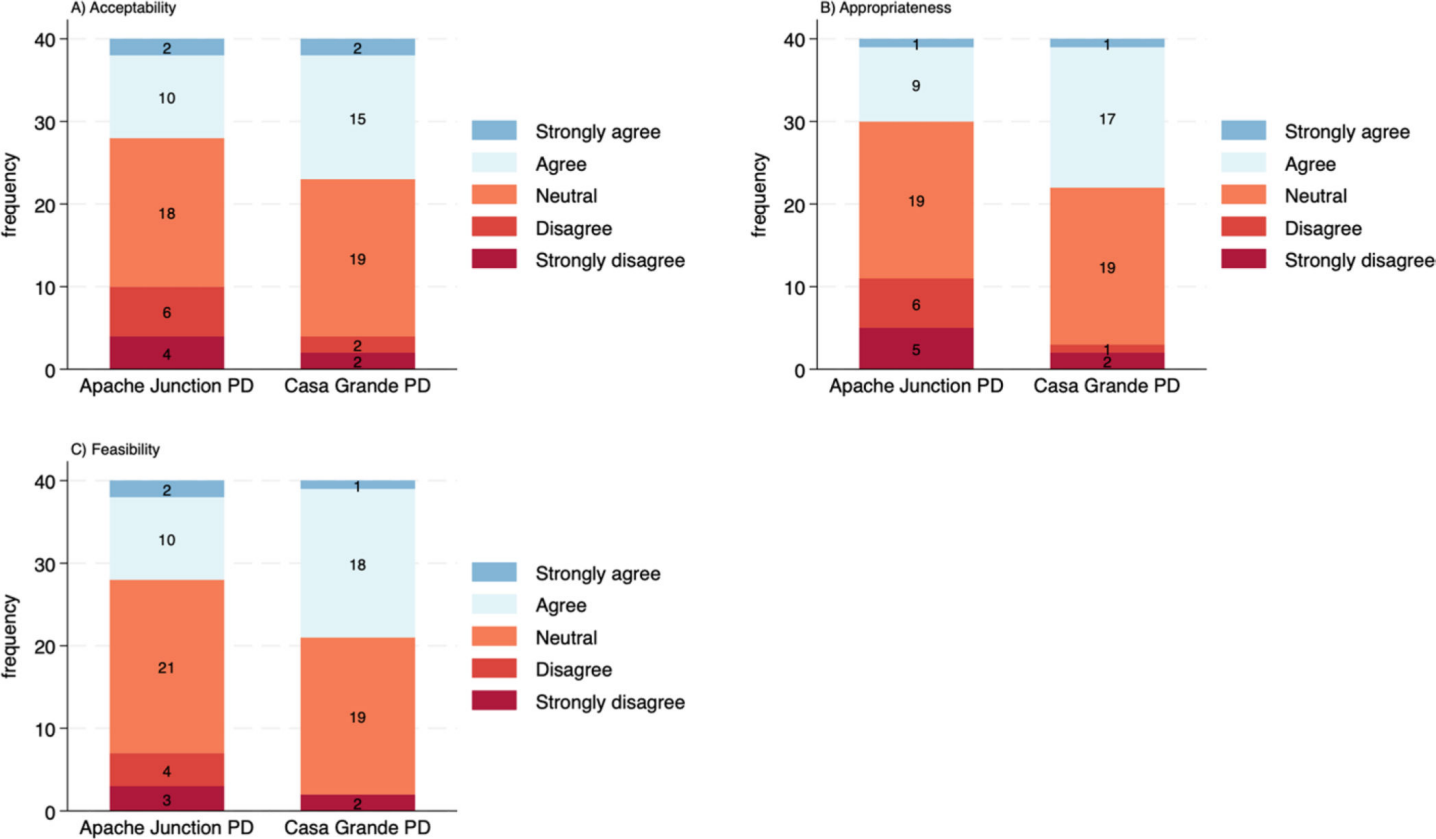
Categorical plots of acceptability, appropriateness, and feasibility

Table 1 also shows officers in CGPD had more positive views of Truleo than their counterparts in AJPD.^6^ Specifically, CGPD officers viewed the platform as more acceptable (3.325 [AJPD = 3.000]), appropriate (3.350 [AJPD = 2.875]), and feasible (3.400 [AJPD = 3.100]). Figure 1 visually portrays these differences in frontline users’ perceived acceptability, appropriateness, and feasibility of the Truleo platform. Likewise, CGPD has fewer respondents who disagreed or strongly disagreed that the Truleo platform was acceptable, appropriate, and feasible. Together, both Table 1; Fig. 1 provide cursory evidence that, after using Truleo for approximately five months, CGPD officers appear to have viewed the platform more positively.

### Ordered logistic regression models

**Fig. 2:**
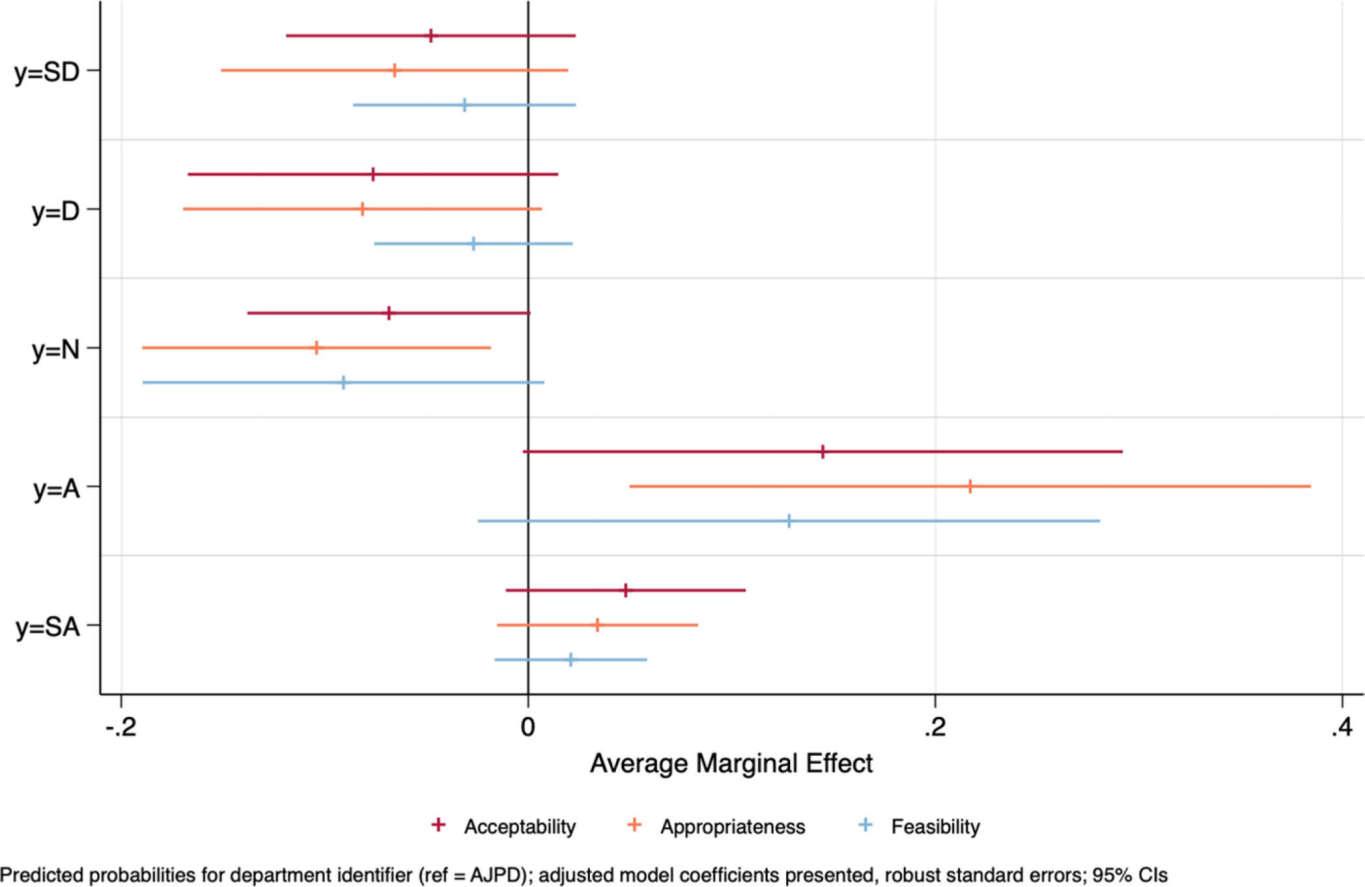
Predicted probabilities for CGPD on acceptability, appropriateness, and feasibility

Table 2 provides the findings from our main ordered logistic regression models.^7^ The coefficients in the table are presented as average marginal effects (AMEs). Though not statistically significant, in the covariate adjusted model CGPD respondents were approximately 14.5%-points more likely to agree that the Truleo platform was acceptable when holding covariates constant (compared to AJPD respondents; AME_adj_ = 0.145, *p* = .054). For our second outcome, appropriateness, CGPD frontline users were less likely to be neutral (AME_unadj_ = −0.076, *p* = .045; AME_adj_ = −0.104, *p* = .017), and more likely to agree Truleo is appropriate (AME_unadj_ = 0.197, *p* = .010; AME_adj_ = 0.217, *p* = .011). The covariate adjusted AME suggests that CGPD respondents, compared to AJPD, were approximately 22%-points more likely to agree that the Truleo platform was appropriate when holding covariates constant. In terms of feasibility, there was less movement in perceptions among CGPD officers compared to AJPD officers. No statistically significant findings emerged, though the direction of the coefficients mirror the prior two models. In alternative specifications which include ordered logistic regression models on collapsed 3-point Likert outcomes and OLS regression models on the 5-point Likert outcomes, similar findings emerged (see Tables A9-A12). Figure 2 provides a coefficient plot to visualize the AMEs for CGPD (compared to AJPD) in the covariate adjusted models.

### Instrumental variable regression models

Lastly, we report the findings from the instrumental variable approach. Table 3 presents the findings from our first stage models, showing that the department identifier is a strong predictor of receiving an email. However, the F-statistic for the first stage models is below the conventional threshold for identifying strong instruments (e.g., F > 10; Stock & Yogo, 2002). The conditional F-statistic (including controls) = 8.88 or 8.94 (depending on the DV). The unconditional F-statistic (excluding covariates) = 18.94. However, because we use robust standard errors (Kaufmann et al., 2019), we also report the Kleibergen-Paap Wald F-statistic = 11.680 or 12.324 (depending on the DV), which is above the conventional threshold of 10. Together, this suggests our instrument is sufficiently strong, though borderline.

In Table 4, our second stage models’ results are presented. In the three second stage models, the instrumented variable (e.g., receiving an email) is significantly associated with an increase in agreement for acceptability (*b* = 1.226, *p* = .042) and appropriateness (*b* = 1.428, *p* = .030). These coefficients suggests that among compliers (e.g., officers in CGPD who report receiving an email), receiving an automated email resulted in an increase of 1.226 for acceptability and 1.428 points for appropriateness. Perceptions of feasibility (*b* = 0.919, *p* = .072) were positive, though just above conventional threshold for significance. Additionally, when holding other variables constant, organizational justice was associated with higher levels of acceptability, appropriateness, and feasibility.

### Summary

We employed a series of analytical approaches to investigate our research questions, including ordered logistic regression models and instrumental variable regression models. In our ordered logistic regression models, CGPD officers were marginally more likely to agree with the acceptability and significantly more likely to agree with the appropriateness of the Truleo platform in the covariate adjusted models. The instrumental variable regression models add further support to the notion that the individual automated emails influenced officer perceptions for appropriateness and acceptability, though only marginally for feasibility. The findings provide evidence that automated emails influenced officers to view the platform as more acceptable and appropriate. These findings are discussed in more detail below.

## Discussion

A critical but understudied component of successful Evidence-Based Policing is evaluating how innovations are implemented, including assessing officers’ acceptance of new innovations. Indeed, the lack of acceptance or buy-in among officers can directly impact implementation and the perceived utility of new technologies, policies, or other reforms. Notably, negative attitudes have hindered the impact of innovations in policing (Bonfine et al., 2014; Formica et al., 2018; Gaub et al., 2016; Terrill & Paoline, 2013; Young & Ready, 2015). Implementation science can help address these issues by evaluating how the implementation impacts the integration of new technologies into organizational operations. However, implementation science has rarely been utilized in police research, especially in the field’s experimental designs (del Pozo et al., 2024). The present study is the first, to our knowledge, to evaluate a natural implementation science experiment in a police setting.

Our findings suggest that automated, AI-generated emails highlighting professionalism plausibly had an impact on officers’ attitudes toward AI-driven BWC review. Both approaches – the ITT and LATE models – suggest that the automated emails were positively associated with officers’ perceptions of the appropriateness and acceptability, and to a lesser extent, the feasibility, of the Truleo platform. Importantly, these findings are within the context of two randomized control trials with similar observed characteristics between respondents from both departments.

The implications of this study’s findings for policing are two-fold. First, the results offer specific insights about the implementation and acceptance of AI-driven body-worn camera analytics. Despite its promise in overcoming a significant BWC review problem (e.g., the 5% problem), prior studies show a fair amount of concern among officers regarding this technology (Adams, 2025), and some departments have experienced significant resistance to its use (Bradbury, 2024; Carter, 2023; Sault, 2023). The results here suggest a means by which the use of such platforms can be more successfully implemented: to provide praise as well as oversight and accountability.

Specifically, the automated feedback feature of Truleo taps into a longstanding problem facing police organizations: good policing is largely invisible and, and as such, is rarely recognized (Fyfe, 1988). This is true both internally and externally – it is difficult to systematically capture officer behavior in ways that adequately measure and praise good police work (Kane & White, 2009). Skolnick and Fyfe (1993: p. 127) highlighted this point in clear terms:

Good cops always seem to identify the causes of problems and to come up with the least troublesome ways of solving them. Good cops think ahead and always leave a way out of any tough situation. Good cops rarely have to resort to the law to solve minor order-maintenance problems like drunks and noisy kids on the street. Good cops spend their time finding out about the people and places on their beats instead of lurking at speed traps or near badly marked stop signs.

Moreover, most measures of police performance involve counts of activities such as stops, confiscating drugs or weapons, arrests, uses of force, complaints – what (Skolnick & Fyfe, 1993) call the “numbers game.” As the quote above illustrates, good policing often doesn’t generate a number.

The inability to identify exceptional police work is amplified by the traditionally punitive philosophy associated with an authoritarian, quasi-military organizational structure (Bittner, 1995). More specifically, the datapoints that are easiest to systematically identify and categorize generally have the potential for negative implications (e.g., misconduct, citizen complaints, uses of force, arrests). Alternatively, it is hard for agencies to accurately search out and quantify positive behaviors that are more subjective such as professionalism or de-escalation attempts, for instance. AI-driven BWC review offers the means to achieve this end by providing a mechanism for line-level officers to not only see their highly professional encounters but also receive recognition for their good work in near real time.

Also worth mentioning is the positive relationship between organizational justice and perceptions of Truleo’s platform. Given the reported cases of resistance to AI within police departments (Carter, 2023; Sault, 2023), it is crucial to consider the level of fairness, neutrality, and respectfulness within police departments as protective factors for the acceptance and diffusion of AI-based technology. In other words, officers who view their employer as fair, unbiased, and respectful are more likely to accept innovations like AI. This finding bodes well with prior literature on the effects of organizational justice within police departments and criminal justice more broadly (Nix & Wolfe, 2016; Wolfe & Lawson, 2020; Wolfe & Piquero, 2011).

Second, the study results have implications for the use of implementation science in policing. By measuring implementation outcomes as variables distinct from the assessment of an innovation’s effectiveness, this study offers a novel demonstration of IS experimentation in a police setting. It introduces the field to the concept of discrete implementation strategies and outcomes (Proctor et al., 2011, 2023), and utilizes psychometric items validated in health care services delivery settings to take their measure (Shea et al., 2014). In doing so, the study demonstrates how the inner setting of an innovation, i.e., the agency in which it is implemented, has variables that can act as barriers and facilitators of organizational change (CFIR, 2023), and illustrates how an experiment involving AI-driven audits of BWC footage and positive feedback can affect police officers’ beliefs about the innovation. Given that even successful, evidence-based changes can fail if they are protested, rejected, or met with resistance by the line-level employees of any organization (del Pozo et al., 2024), this study helps police researchers concerned with the update of evidence-based practices operationalize such variables and design prospective experiments to assess which strategies produce the best implementation outcomes (see Bauer et al., 2015). Future research can use the Consolidated Framework for Implementation Research (CFIR, 2023) as an inventory of constructs and variables that can be adapted to police settings and experimentally tested.

## Limitations

Our study has limitations that warrant discussion. First, the study focuses on two mid-sized police departments in the Phoenix metropolitan area, which limits the generalizability of results to other police departments, especially in other regions of the United States or beyond. Second, the data comes from cross-sectional surveys which (a) only capture perceptions at a specific point in time, not over time and (b) can be subject to some reporting error. Relatedly, given we ask specifically, “Have you received an email from Truleo praising you for a “highly professional” encounter?”, it is possible that there is some measurement error around the responses for this question because there are at least two ways an officer could be notified of their highly professional encounters: (1) Truleo’s individual automated emails regarding high professionalism – the focus of the present study or (2) an immediate supervisor sends a “praise” notification regarding an officer’s encounter through the Truleo platform. If this latter method was utilized more in CGPD than in AJPD, it could plausibly confound the results we attributed to the use of Truleo’s automated feedback. We attempt to mitigate this potential confounding pathway by including a measure of perceived supervisor support.

Fourth, it is possible that unobserved department culture or leadership factors in the CGPD yielded ample positive messaging about the Truleo platform throughout the study period, thereby influencing frontline users’ perceptions in a way that confounds the effects observed here. We did attempt to address this by including perceived organizational justice in our regression models. It is worth noting, however, that in the pre-intervention (see Watts et al., 2024) and post-intervention focus groups with leadership at both departments, both expressed positive attitudes towards the technology and have continued with its use following their respective 6-month RCT study periods. This, suggests that, qualitatively speaking, leadership in both departments thought highly of the platform throughout the 6-month study period, and we do not have cause to believe that one was more vocal about this enthusiasm than the other. Fifth, our primary dependent variables were not observed in the pre-period survey. Thus, we do not have an apples-to-apples comparison in terms of a pre-post comparison of perceptions for our outcomes. We mitigate this issue by selecting highly correlated outcomes that capture respondents’ perceptions of Truleo which show there are no statically significant differences at baseline. Additionally, 17 (42.5%) officers in AJPD report receiving an email despite the automated email feature being disabled. As discussed above, it is possible that AJPD officers reported the supervisor generated praise notifications as the automated emails leading to AJPD officers responding in the affirmative in our survey. This does present some challenges to the exclusion restriction for our instrumental variable models (Angrist et al., 1996), meaning our estimates may be attenuated to some extent. Further, our instrumental variable is on the weaker side in terms of conventional F-statistics, though not excessively so. The Kleibergen-Paap statistic presented in our Results suggests, however, that our instrument is sufficiently strong though borderline.

Lastly, it is also important to note that this study was not an effectiveness trial of Truleo, i.e., it does not purport to assess whether the platform accurately identifies and categorizes the police behaviors of interest, and whether this has the net effect of improving police professionalism and other aspects of job performance. These are *effectiveness* outcomes, and we have endeavored to study the *implementation* outcomes of the feasibility, acceptability, and appropriateness of the product under study, specifically, whether AI-generated positive feedback significantly affects these independent implementation outcome variables. Importantly, police departments adopt this technology as is, thus it is important to evaluate it within that context. Should future research determine that Truleo is inaccurate in its measurements or promotes substandard behaviors by incorrectly categorizing them as desirable ones, it would be evidence that the platform was ineffective, even if its automated positive feedback processes improved the likelihood that it could be successfully implemented in agencies.

## Conclusion

Implementation science offers a unique and underutilized framework to evaluate *how* the implementation of evidence-based practices impact integration into operations and the subsequent effectiveness of that practice. The current study used this framework to better understand how automated positive reinforcement emails created and delivered through an AI-driven BWC analytics platform, Truleo, impacted officers’ beliefs about the platform. Importantly, positive reinforcement mechanisms are often overlooked in the world of policing due to the quasi-militaristic, punitive, and liability-oriented nature of the profession. Incorporating positive reinforcement addresses a longstanding deficiency in policing: identifying and praising good police work. The findings of the present study provide evidence that individual automated emails influenced officers’ perceptions in CGPD, leading them to view the AI-driven BWC platform as more acceptable and appropriate than in the agency that did not utilize this automated feedback function. These findings reinforce the idea that *how* technological change is implemented in policing can shape the success of an initiative regardless of whether the technology is inherently effective or valuable. The findings therefore shed light on an important and understudied area in the profession: the various methods agencies can use to implement technological change, and how these methods shape frontline users’ attitudes toward the cultural and organizational changes these technologies will usher in.

## Supplementary Material

supplementary material

**Supplementary Information** The online version contains supplementary material available at https://doi.org/10.1007/s11292-025-09711-7.

## Figures and Tables

**Table 1:** Summary statistics

	Apache Junction PD *N* = 43				Casa Grande PD *N* = 41			

	Mean/%	SD	Min	Max	Mean/%	SD	Min	Max
*Dependent Variables* ^a^								
Acceptability	3.00	1.01	1.00	5.00	3.33	0.86	1.00	5.00
Appropriateness	2.88	0.99	1.00	5.00	3.35	0.80	1.00	5.00
Feasibility	3.10	0.93	1.00	5.00	3.40	0.78	1.00	5.00
Organizational justice^b^	−0.14	0.88	−2.65	1.95	−0.14	0.82	−2.85	1.35
Perceived supervisor support^b^	−0.20	1.30	−3.04	1.21	−0.11	0.94	−3.04	1.21
Received highly professional email	0.42	-	0.00	1.00	0.85	-	0.00	1.00
Treatment assignment	0.38	-	0.00	1.00	0.46	-	0.00	1.00
Patrol	0.58	-	0.00	1.00	0.73	-	0.00	1.00
Female	0.13	-	0.00	1.00	0.10	-	0.00	1.00
Age	38.69	10.30	21.00	61.00	36.11	8.78	24.00	56.00
White	0.64	-	0.00	1.00	0.54	-	0.00	1.00
Black	0.05	-	0.00	1.00	0.10	-	0.00	1.00
Hispanic	0.13	-	0.00	1.00	0.28	-	0.00	1.00
Other	0.18	-	0.00	1.00	0.08	-	0.00	1.00
Bachelor’s degree or higher	0.30	-	0.00	1.00	0.21	-	0.00	1.00

a 5-point Likert scale (1 = Strongly disagree, 3 = Neutral, 5 = Strongly agree)

b Standardized (mean = 0, SD = 1)

**Table 2:** Ordered logistic regression models predicting acceptability, appropriateness, feasibility

Acceptability										

	y = SD		y = D		y = N		y =A		y = SA	
	Unadjusted	Adjusted	Unadjusted	Adjusted	Unadjusted	Adjusted	Unadjusted	Adjusted	Unadjusted	Adjusted
CGPD (ref = AJPD)	−0.046	−0.048	−0.049	−0.076	−0.055	−0.068	0.119	0.145	0.031	0.048
	(0.034)	(0.036)	(0.036)	(0.046)	(0.036)	(0.036)	(0.073)	(0.075)	(0.023)	(0.030)
	[0.180]	[0.188]	[0.168]	[0.101]	[0.126]	[0.054]	[0.106]	[0.054]	[0.179]	[0.112]
Observations	80	75	80	75	80	75	80	75	80	75
Appropriateness										
CGPD (ref = AJPD)	−0.082	−0.066	−0.064	−0.081	−0.076*	−0.104*	0.197*	0.217*	0.025	0.034
	(0.044)	(0.044)	(0.035)	(0.045)	(0.038)	(0.044)	(0.076)	(0.085)	(0.019)	(0.025)
	[0.061]	[0.131]	[0.069]	[0.070]	[0.045]	[0.017]	[0.010]	[0.011]	[0.187]	[0.177]
Observations	80	76	80	76	80	76	80	76	80	76
Feasibility										
CGPD (ref = AJPD)	−0.045	−0.031	−0.032	−0.027	−0.101	−0.091	0.150	0.128	0.028	0.021
	(0.031)	(0.028)	(0.026)	(0.025)	(0.053)	(0.050)	(0.083)	(0.078)	(0.019)	(0.019)
	[0.141]	[0.263]	[0.219]	[0.280]	[0.056]	[0.071]	[0.070]	[0.101]	[0.145]	[0.274]
Observations	80	76	80	76	80	76	80	76	80	76

Average marginal effects (AMEs) presented, robust standard errors, p-value in brackets; Adjusted models control for RCT assignment, organizational justice, perceived supervisor support, patrol, gender, race, age, education but are not shown; SD = Strongly disagree, D = Disagree, N = Neutral, A = Agree, SA = Strongly agree;

* p>.05

** p>.01

*** p>.001

**Table 3:** First stage IV models predicting email received

	(DV: Acceptability)	(DV: Appropriateness)	(DV: Feasibility)

CGPD (ref = AJPD)	0.368**	0.372***	0.372***
	(0.108)	(0.106)	(0.106)
	[0.001]	[0.001]	[0.001]
Treatment assignment	0.167	0.174	0.174
	(0.106)	(0.104)	(0.104)
	[0.120]	[0.099]	[0.099]
Organizational justice	−0.112	−0.108	−0.108
	(0.096)	(0.096)	(0.096)
	[0.251]	[0.264]	[0.264]
Perceived supervisor support	0.086	0.083	0.083
	(0.052)	(0.052)	(0.052)
	[0.105]	[0.112]	[0.112]
Patrol	0.261	0.255	0.255
	(0.137)	(0.135)	(0.135)
	[0.062]	[0.063]	[0.063]
Age	0.005	0.005	0.005
	(0.005)	(0.005)	(0.005)
	[0.351]	[0.316]	[0.316]
Female	−0.048	−0.053	−0.053
	(0.176)	(0.174)	(0.174)
	[0.787]	[0.763]	[0.763]
Black	0.012	0.012	0.012
	(0.158)	(0.159)	(0.159)
	[0.938]	[0.939]	[0.939]
Hispanic	0.028	0.040	0.040
	(0.116)	(0.111)	(0.111)
	[0.813]	[0.719]	[0.719]
Other	−0.204	−0.205	−0.205
	(0.166)	(0.166)	(0.166)
	[0.222]	[0.221]	[0.221]
Bachelor’s degree or higher	−0.149	−0.151	−0.151
	(0.138)	(0.138)	(0.138)
	[0.287]	[0.278]	[0.278]
Constant	0.080	0.068	0.068
	(0.276)	(0.276)	(0.276)
	[0.772]	[0.806]	[0.806]
Observations	75	76	76
R2	0.387	0.391	0.391
RMSE	0.416	0.413	0.413
F	8.944	8.888	8.888
Kleibergen-Paap F	11.680	12.324	12.324

First stage OLS regression predicting receiving an email; robust standard errors; p-values in brackets

* *p* <.05

** *p* <.01

*** *p* <.001

**Table 4:** Second stage IV models

	(1)	(2)	(3)

	Acceptability	Appropriateness	Feasibility
Email (instrumented)	1.226*	1.428*	0.919
	(0.603)	(0.657)	(0.511)
	[0.042]	[0.030]	[0.072]
Treatment assignment	−0.029	−0.063	0.133
	(0.276)	(0.280)	(0.253)
	[0.916]	[0.822]	[0.600]
Organizational justice	0.454*	0.420*	0.326*
	(0.201)	(0.194)	(0.149)
	[0.024]	[0.030]	[0.029]
Perceived supervisor support	−0.037	−0.038	0.064
	(0.143)	(0.147)	(0.111)
	[0.797]	[0.797]	[0.562]
Patrol	−0.402	−0.604	−0.367
	(0.286)	(0.317)	(0.247)
	[0.159]	[0.057]	[0.137]
Age	−0.003	−0.010	−0.010
	(0.012)	(0.012)	(0.010)
	[0.805]	[0.406]	[0.319]
Female	0.389	0.448	0.281
	(0.270)	(0.288)	(0.256)
	[0.149]	[0.120]	[0.271]
Black	0.103	−0.054	0.192
	(0.312)	(0.333)	(0.257)
	[0.741]	[0.871]	[0.456]
Hispanic	0.174	−0.243	0.210
	(0.298)	(0.289)	(0.227)
	[0.560]	[0.401]	[0.354]
Other	0.293	−0.029	0.083
	(0.373)	(0.361)	(0.299)
	[0.433]	[0.936]	[0.781]
Bachelor’s degree or higher	0.457	0.463	0.294
	(0.296)	(0.259)	(0.274)
	[0.123]	[0.074]	[0.284]
Constant	2.636***	3.031***	3.144***
	(0.597)	(0.571)	(0.515)
	[0.000]	[0.000]	[0.000]
Observations	75	76	76
RMSE	0.860	0.877	0.732

Second stage models are OLS regression models (2SLS); robust standard errors in parentheses

*p*-values in brackets

* *p* <.05

** *p* <.01

*** *p* <.001

## Data Availability

Data and replication code are available at OSF: https://osf.io/b2jc7/files/osfstorage.

## Footnotes

1 See https://help.truleo.co/how-truleo-recognizes-professionalism.

2 In the 6-month RCT for the two departments, out of approximately 40,000 encounters about 5–10% of encounters were labeled as high professionalism. The vast majority (90–95%) were rated as standard professionalism. Just 2 encounters were labeled as substandard professionalism (< 1%).

3 The same survey was administered in the pre-intervention period, and the findings are discussed in depth elsewhere (Watts et al., 2024). The data we use in the present study focuses on post-intervention responses. We use the same data as White et al. (2025) where they look at differences from pre- to post-intervention and between treatment and control groups, though they do not look at the implementation science outcomes which are the focus of the present study.

4 To capture treatment assignment in our survey, we ask respondents to respond “yes” or “no” to the question “Since [RCT start date] (Truleo’s start date at the dept.) have you had access to your Truleo account?”

5 Following the administration of our pre-intervention surveys in both departments we decided to incorporate items from Weiner et al. (2017) in the post-intervention survey to try and capture how the varying methods of implementation (enabling automated emails from Truleo) impacts officers’ perceptions. Thus, in the pre-intervention surveys, the direct items were not present.

6 See Table A1 for a breakdown by RCT treatment assignment.

7 Full models including control variable coefficients are in the supplemental file (Tables A6 – A8).
