# Supplementary material for: Does automated feedback impact the acceptability of AI-generated police body worn camera review? An implementation science natural experiment
Source: J Exp Criminol. Author manuscript; Available in PMC 2026 Jan 14. (PMC12798756; doi:10.1007/s11292-025-09711-7)
Supplement: supplementary material [file NIHMS2125337-supplement-supplementary_material.docx]

**Appendix**

| Table A1. Descriptive Statistics: AJPD and CGPD by RCT assignment | | | | | | | | |
| --- | --- | --- | --- | --- | --- | --- | --- | --- |
|  | *Automated email disabled* | | | | *Automated email enabled* | | | |
|  | AJPD Control  N=25 | | AJPD TX  N=15 | | CGPD Control  N=22 | | CGPD TX  N=19 | |
|  | Mean/% | SD | Mean/% | SD | Mean/% | SD | Mean/% | SD |
| Acceptability^a^ | 2.88 | 0.83 | 3.20 | 1.26 | 3.36 | 0.79 | 3.28 | 0.96 |
| Appropriateness^a^ | 2.76 | 0.88 | 3.07 | 1.16 | 3.36 | 0.79 | 3.33 | 0.84 |
| Feasibility^a^ | 3.12 | 0.73 | 3.07 | 1.22 | 3.27 | 0.77 | 3.56 | 0.78 |
| Organizational justice^b^ | -0.00 | 0.84 | -0.36 | 0.92 | -0.12 | 0.84 | -0.16 | 0.83 |
| Perceived supervisor support^b^ | -0.10 | 1.12 | -0.35 | 1.58 | -0.21 | 1.15 | 0.02 | 0.60 |
| Received highly professional email | 0.24 | - | 0.73 | - | 0.86 | - | 0.84 | - |
| Patrol | 0.44 | - | 0.80 | - | 0.77 | - | 0.68 | - |
| Female | 0.12 | - | 0.14 | - | 0.10 | - | 0.11 | - |
| Age | 38.80 | 9.64 | 38.50 | 11.77 | 33.71 | 7.29 | 39.06 | 9.74 |
| White | 0.54 | - | 0.80 | - | 0.52 | - | 0.56 | - |
| Black | 0.08 | - | 0.00 | - | 0.14 | - | 0.06 | - |
| Hispanic | 0.17 | - | 0.07 | - | 0.29 | - | 0.28 | - |
| Other | 0.21 | - | 0.13 | - | 0.05 | - | 0.11 | - |
| Bachelor's degree or higher | 0.32 | - | 0.27 | - | 0.19 | - | 0.24 | - |

^a^5-point Likert scale (1 = Strongly disagree, 3 = Neutral, 5 = Strongly agree); ^b^Standardized (mean = 0, SD = 1).

Table A2. Demographics Among Officers Pre-period: T-tests

|  |  |  |  |  |
| --- | --- | --- | --- | --- |
|  | AJPD  N = 32 | CGPD  N = 49 | Delta | P-value |
| Patrol | 0.69 | 0.76 | -0.068 | 0.510 |
| Female | 0.10 | 0.09 | 0.016 | 0.814 |
| Age | 38.50 | 36.78 | 1.722 | 0.466 |
| White | 0.69 | 0.57 | 0.115 | 0.322 |
| Black | 0.07 | 0.04 | 0.026 | 0.622 |
| Other | 0.14 | 0.06 | 0.074 | 0.284 |
| Bachelor's degree or higher | 0.33 | 0.28 | 0.057 | 0.601 |

Table A3. Demographics Among Officers Post-period: T-tests

|  |  |  |  |  |
| --- | --- | --- | --- | --- |
|  | AJPD  N = 43 | CGPD  N = 41 | Delta | P-value |
| Patrol | 0.58 | 0.73 | -0.150 | 0.151 |
| Female | 0.13 | 0.10 | 0.029 | 0.697 |
| Age | 38.69 | 36.11 | 2.587 | 0.240 |
| White | 0.64 | 0.54 | 0.103 | 0.364 |
| Black | 0.05 | 0.10 | -0.051 | 0.402 |
| Other | 0.18 | 0.08 | 0.103 | 0.180 |
| Bachelor's degree or higher | 0.30 | 0.21 | 0.089 | 0.372 |

Table A4. Demographics Among Officers: Pre- and Post-period

|  | Pooled (n = 165) | | | | Apache Junction PD (n = 75) | | | | Casa Grande PD (n = 90) | | | |
| --- | --- | --- | --- | --- | --- | --- | --- | --- | --- | --- | --- | --- |
|  | Pre | Post | Delta | P-value | Pre | Post | Delta | P-value | Pre | Post | Delta | P-value |
| Patrol | 0.73 | 0.65 | 0.074 | 0.309 | 0.69 | 0.58 | 0.106 | 0.354 | 0.76 | 0.73 | 0.023 | 0.803 |
| Female | 0.09 | 0.12 | -0.024 | 0.639 | 0.10 | 0.13 | -0.028 | 0.730 | 0.09 | 0.10 | -0.016 | 0.809 |
| Age | 37.44 | 37.42 | 0.023 | 0.989 | 38.50 | 38.69 | -0.192 | 0.936 | 36.78 | 36.11 | 0.673 | 0.753 |
| White | 0.62 | 0.59 | 0.029 | 0.718 | 0.69 | 0.64 | 0.049 | 0.681 | 0.57 | 0.54 | 0.036 | 0.741 |
| Black | 0.05 | 0.08 | -0.024 | 0.544 | 0.07 | 0.05 | 0.018 | 0.763 | 0.04 | 0.10 | -0.060 | 0.282 |
| Other | 0.09 | 0.13 | -0.036 | 0.478 | 0.14 | 0.18 | -0.042 | 0.651 | 0.06 | 0.08 | -0.013 | 0.815 |
| Bachelor's degree or higher | 0.30 | 0.26 | 0.042 | 0.560 | 0.33 | 0.30 | 0.033 | 0.770 | 0.28 | 0.21 | 0.066 | 0.488 |

|  |  |
| --- | --- |

Table A5. Perceptions of DV Proxies by Department Among Officers: Pre-period Balance

|  |  |  |  |  |  |
| --- | --- | --- | --- | --- | --- |
|  | AJPD  N = 32 | CGPD  N = 49 | Delta | P-value | Pearson Correlation Coefficient |
| [Truleo] Will help improve how I interact with the public. | 2.38 | 2.67 | -0.298 | 0.117 | Acceptability r = .60, Appropriateness r = .54, Feasibility r = .54 |
| [Truleo] Will help justify my on-duty actions. | 2.78 | 3.12 | -0.341 | 0.117 | Acceptability r = .59, Appropriateness r = .64, Feasibility r = .51 |
| [Truleo] Will improve how my colleagues interact with the public. | 2.81 | 2.88 | -0.065 | 0.744 | Acceptability r = .59, Appropriateness r = .63, Feasibility r = .56 |
| Overall, [Truleo] is a valuable addition to my work. | 2.62 | 2.80 | -0.171 | 0.467 | Acceptability r = .76,  Appropriateness r = .74, Feasibility r = .69 |
| I am excited to see how my performance is captured by Truleo. | 2.75 | 2.86 | -0.107 | 0.660 | Acceptability r = .71, Appropriateness r = .66, Feasibility r = .61 |
| [Truleo] Will improve the department's performance evaluation process. | 2.81 | 2.90 | -0.085 | 0.671 | Acceptability r = .66, Appropriateness r = .73, Feasibility r = .57 |
| [Truleo] Will allow the department to publicize the good work done. | 3.16 | 3.04 | 0.115 | 0.575 | Acceptability r = .73, Appropriateness r = .74, Feasibility r = .59 |

| Table A6. Full Models: Acceptability | | | | | | | | | | |
| --- | --- | --- | --- | --- | --- | --- | --- | --- | --- | --- |
|  | y=SD | | y=D | | y=N | | y=A | | y=SA | |
|  | Unadjusted | Adjusted | Unadjusted | Adjusted | Unadjusted | Adjusted | Unadjusted | Adjusted | Unadjusted | Adjusted |
| CGPD (ref = AJPD) | -0.046 | -0.048 | -0.049 | -0.076 | -0.055 | -0.068 | 0.119 | 0.145 | 0.031 | 0.048 |
|  | (0.034) | (0.036) | (0.036) | (0.046) | (0.036) | (0.036) | (0.073) | (0.075) | (0.023) | (0.030) |
|  | [0.180] | [0.188] | [0.168] | [0.101] | [0.126] | [0.054] | [0.106] | [0.054] | [0.179] | [0.112] |
| Treatment assignment |  | -0.019 |  | -0.031 |  | -0.028 |  | 0.058 |  | 0.019 |
|  |  | (0.029) |  | (0.044) |  | (0.045) |  | (0.085) |  | (0.031) |
|  |  | [0.506] |  | [0.484] |  | [0.541] |  | [0.493] |  | [0.533] |
| Organizational justice |  | -0.037 |  | -0.059 |  | -0.053 |  | 0.111 |  | 0.037 |
|  |  | (0.027) |  | (0.043) |  | (0.042) |  | (0.076) |  | (0.026) |
|  |  | [0.174] |  | [0.170] |  | [0.207] |  | [0.146] |  | [0.165] |
| Perceived supervisor support |  | -0.003 |  | -0.005 |  | -0.004 |  | 0.009 |  | 0.003 |
|  |  | (0.017) |  | (0.027) |  | (0.024) |  | (0.051) |  | (0.017) |
|  |  | [0.864] |  | [0.857] |  | [0.857] |  | [0.858] |  | [0.861] |
| Patrol |  | 0.007 |  | 0.010 |  | 0.009 |  | -0.020 |  | -0.007 |
|  |  | (0.031) |  | (0.048) |  | (0.043) |  | (0.091) |  | (0.030) |
|  |  | [0.832] |  | [0.826] |  | [0.828] |  | [0.828] |  | [0.829] |
| Age |  | -0.000 |  | -0.001 |  | -0.001 |  | 0.001 |  | 0.000 |
|  |  | (0.002) |  | (0.003) |  | (0.002) |  | (0.005) |  | (0.002) |
|  |  | [0.808] |  | [0.806] |  | [0.801] |  | [0.804] |  | [0.805] |
| Female |  | -0.037 |  | -0.059 |  | -0.053 |  | 0.113 |  | 0.037 |
|  |  | (0.044) |  | (0.064) |  | (0.057) |  | (0.120) |  | (0.039) |
|  |  | [0.392] |  | [0.351] |  | [0.345] |  | [0.345] |  | [0.333] |
| Black |  | -0.013 |  | -0.022 |  | -0.019 |  | 0.041 |  | 0.013 |
|  |  | (0.032) |  | (0.057) |  | (0.060) |  | (0.112) |  | (0.037) |
|  |  | [0.682] |  | [0.706] |  | [0.750] |  | [0.712] |  | [0.730] |
| Hispanic |  | -0.023 |  | -0.040 |  | -0.045 |  | 0.080 |  | 0.027 |
|  |  | (0.034) |  | (0.061) |  | (0.093) |  | (0.133) |  | (0.053) |
|  |  | [0.496] |  | [0.519] |  | [0.631] |  | [0.547] |  | [0.605] |
| Other |  | -0.013 |  | -0.022 |  | -0.019 |  | 0.042 |  | 0.013 |
|  |  | (0.036) |  | (0.065) |  | (0.066) |  | (0.126) |  | (0.041) |
|  |  | [0.714] |  | [0.740] |  | [0.769] |  | [0.741] |  | [0.754] |
| Bachelor's degree or higher |  | -0.034 |  | -0.054 |  | -0.048 |  | 0.102 |  | 0.034 |
|  |  | (0.036) |  | (0.064) |  | (0.055) |  | (0.111) |  | (0.039) |
|  |  | [0.347] |  | [0.399] |  | [0.379] |  | [0.356] |  | [0.387] |
| Observations | 80 | 75 | 80 | 75 | 80 | 75 | 80 | 75 | 80 | 75 |

Average marginal effects (AMEs) presented, robust standard errors in parentheses, p-value in brackets; SD = Strongly disagree, D = Disagree, N = Neutral, A = Agree, SA = Strongly agree.

| Table A7. Full Models: Appropriateness | | | | | | | | | | |
| --- | --- | --- | --- | --- | --- | --- | --- | --- | --- | --- |
|  | y=SD | | y=D | | y=N | | y=A | | y=SA | |
|  | Unadjusted | Adjusted | Unadjusted | Adjusted | Unadjusted | Adjusted | Unadjusted | Adjusted | Unadjusted | Adjusted |
| CGPD (ref = AJPD) | -0.082 | -0.066 | -0.064 | -0.081 | -0.076 | -0.104 | 0.197 | 0.217 | 0.025 | 0.034 |
|  | (0.044) | (0.044) | (0.035) | (0.045) | (0.038) | (0.044) | (0.076) | (0.085) | (0.019) | (0.025) |
|  | [0.061] | [0.131] | [0.069] | [0.070] | [0.045] | [0.017] | [0.010] | [0.011] | [0.187] | [0.177] |
| Treatment assignment |  | -0.030 |  | -0.038 |  | -0.048 |  | 0.100 |  | 0.016 |
|  |  | (0.028) |  | (0.035) |  | (0.048) |  | (0.089) |  | (0.017) |
|  |  | [0.280] |  | [0.283] |  | [0.317] |  | [0.261] |  | [0.350] |
| Organizational justice |  | -0.030 |  | -0.037 |  | -0.047 |  | 0.098 |  | 0.015 |
|  |  | (0.023) |  | (0.026) |  | (0.033) |  | (0.064) |  | (0.012) |
|  |  | [0.201] |  | [0.157] |  | [0.159] |  | [0.123] |  | [0.218] |
| Perceived supervisor support |  | -0.008 |  | -0.010 |  | -0.013 |  | 0.028 |  | 0.004 |
|  |  | (0.016) |  | (0.019) |  | (0.024) |  | (0.049) |  | (0.009) |
|  |  | [0.591] |  | [0.581] |  | [0.580] |  | [0.569] |  | [0.629] |
| Patrol |  | 0.033 |  | 0.041 |  | 0.053 |  | -0.110 |  | -0.017 |
|  |  | (0.032) |  | (0.035) |  | (0.044) |  | (0.088) |  | (0.017) |
|  |  | [0.301] |  | [0.239] |  | [0.230] |  | [0.211] |  | [0.317] |
| Age |  | 0.000 |  | 0.000 |  | 0.000 |  | -0.001 |  | -0.000 |
|  |  | (0.001) |  | (0.002) |  | (0.002) |  | (0.005) |  | (0.001) |
|  |  | [0.874] |  | [0.874] |  | [0.876] |  | [0.874] |  | [0.878] |
| Female |  | -0.046 |  | -0.056 |  | -0.072 |  | 0.151 |  | 0.024 |
|  |  | (0.045) |  | (0.049) |  | (0.062) |  | (0.125) |  | (0.023) |
|  |  | [0.313] |  | [0.249] |  | [0.245] |  | [0.229] |  | [0.308] |
| Black |  | 0.003 |  | 0.003 |  | 0.005 |  | -0.010 |  | -0.002 |
|  |  | (0.034) |  | (0.045) |  | (0.067) |  | (0.125) |  | (0.021) |
|  |  | [0.941] |  | [0.940] |  | [0.938] |  | [0.939] |  | [0.939] |
| Hispanic |  | 0.010 |  | 0.013 |  | 0.018 |  | -0.036 |  | -0.006 |
|  |  | (0.038) |  | (0.045) |  | (0.054) |  | (0.117) |  | (0.019) |
|  |  | [0.785] |  | [0.772] |  | [0.744] |  | [0.761] |  | [0.776] |
| Other |  | 0.040 |  | 0.045 |  | 0.038 |  | -0.108 |  | -0.014 |
|  |  | (0.043) |  | (0.038) |  | (0.034) |  | (0.088) |  | (0.017) |
|  |  | [0.351] |  | [0.243] |  | [0.263] |  | [0.222] |  | [0.398] |
| Bachelor's degree or higher |  | -0.038 |  | -0.047 |  | -0.060 |  | 0.125 |  | 0.020 |
|  |  | (0.036) |  | (0.039) |  | (0.047) |  | (0.100) |  | (0.014) |
|  |  | [0.289] |  | [0.231] |  | [0.198] |  | [0.213] |  | [0.172] |
| Observations | 80 | 76 | 80 | 76 | 80 | 76 | 80 | 76 | 80 | 76 |

Average marginal effects (AMEs) presented, robust standard errors in parentheses, p-value in brackets; SD = Strongly disagree, D = Disagree, N = Neutral, A = Agree, SA = Strongly agree.

| Table A8. Full Models: Feasibility | | | | | | | | | | |
| --- | --- | --- | --- | --- | --- | --- | --- | --- | --- | --- |
|  | y=SD | | y=D | | y=N | | y=A | | y=SA | |
|  | Unadjusted | Adjusted | Unadjusted | Adjusted | Unadjusted | Adjusted | Unadjusted | Adjusted | Unadjusted | Adjusted |
| CGPD (ref = AJPD) | -0.045 | -0.031 | -0.032 | -0.027 | -0.101 | -0.091 | 0.150 | 0.128 | 0.028 | 0.021 |
|  | (0.031) | (0.028) | (0.026) | (0.025) | (0.053) | (0.050) | (0.083) | (0.078) | (0.019) | (0.019) |
|  | [0.141] | [0.263] | [0.219] | [0.280] | [0.056] | [0.071] | [0.070] | [0.101] | [0.145] | [0.274] |
| Treatment assignment |  | -0.051 |  | -0.044 |  | -0.148 |  | 0.209 |  | 0.034 |
|  |  | (0.032) |  | (0.026) |  | (0.091) |  | (0.107) |  | (0.025) |
|  |  | [0.106] |  | [0.094] |  | [0.106] |  | [0.052] |  | [0.177] |
| Organizational justice |  | -0.032 |  | -0.027 |  | -0.093 |  | 0.131 |  | 0.021 |
|  |  | (0.023) |  | (0.019) |  | (0.053) |  | (0.070) |  | (0.016) |
|  |  | [0.161] |  | [0.155] |  | [0.083] |  | [0.061] |  | [0.185] |
| Perceived supervisor support |  | -0.011 |  | -0.010 |  | -0.033 |  | 0.046 |  | 0.008 |
|  |  | (0.017) |  | (0.011) |  | (0.033) |  | (0.050) |  | (0.010) |
|  |  | [0.496] |  | [0.378] |  | [0.318] |  | [0.351] |  | [0.439] |
| Patrol |  | 0.016 |  | 0.014 |  | 0.046 |  | -0.064 |  | -0.011 |
|  |  | (0.026) |  | (0.020) |  | (0.068) |  | (0.096) |  | (0.017) |
|  |  | [0.547] |  | [0.505] |  | [0.503] |  | [0.501] |  | [0.537] |
| Age |  | 0.001 |  | 0.001 |  | 0.003 |  | -0.004 |  | -0.001 |
|  |  | (0.001) |  | (0.001) |  | (0.004) |  | (0.006) |  | (0.001) |
|  |  | [0.506] |  | [0.494] |  | [0.484] |  | [0.480] |  | [0.500] |
| Female |  | -0.027 |  | -0.023 |  | -0.078 |  | 0.111 |  | 0.018 |
|  |  | (0.045) |  | (0.033) |  | (0.119) |  | (0.166) |  | (0.028) |
|  |  | [0.546] |  | [0.486] |  | [0.509] |  | [0.504] |  | [0.526] |
| Black |  | -0.020 |  | -0.019 |  | -0.098 |  | 0.118 |  | 0.020 |
|  |  | (0.021) |  | (0.023) |  | (0.139) |  | (0.147) |  | (0.032) |
|  |  | [0.326] |  | [0.405] |  | [0.479] |  | [0.422] |  | [0.537] |
| Hispanic |  | -0.020 |  | -0.019 |  | -0.094 |  | 0.114 |  | 0.019 |
|  |  | (0.020) |  | (0.023) |  | (0.121) |  | (0.134) |  | (0.026) |
|  |  | [0.313] |  | [0.421] |  | [0.435] |  | [0.394] |  | [0.481] |
| Other |  | 0.031 |  | 0.024 |  | 0.051 |  | -0.098 |  | -0.009 |
|  |  | (0.043) |  | (0.026) |  | (0.055) |  | (0.107) |  | (0.011) |
|  |  | [0.460] |  | [0.348] |  | [0.349] |  | [0.359] |  | [0.418] |
| Bachelor's degree or higher |  | -0.014 |  | -0.012 |  | -0.042 |  | 0.059 |  | 0.010 |
|  |  | (0.029) |  | (0.028) |  | (0.089) |  | (0.122) |  | (0.023) |
|  |  | [0.623] |  | [0.663] |  | [0.641] |  | [0.631] |  | [0.681] |
| Observations | 80 | 76 | 80 | 76 | 80 | 76 | 80 | 76 | 80 | 76 |

Average marginal effects (AMEs) presented, robust standard errors in parentheses, p-value in brackets; SD = Strongly disagree, D = Disagree, N = Neutral, A = Agree, SA = Strongly agree.

| Table A9. 3-point Ordered Logistic Regression: Acceptability | | | |
| --- | --- | --- | --- |
|  | y=D | y=N | y=A |
|  | b/se/p | b/se/p | b/se/p |
| CGPD (ref = AJPD) | -0.128 | -0.077 | 0.205 |
|  | (0.071) | (0.039) | (0.094) |
|  | [0.071] | [0.047] | [0.029] |
| Treatment assignment | -0.050 | -0.030 | 0.080 |
|  | (0.065) | (0.044) | (0.107) |
|  | [0.442] | [0.488] | [0.451] |
| Organizational justice | -0.115 | -0.069 | 0.184 |
|  | (0.062) | (0.044) | (0.093) |
|  | [0.064] | [0.117] | [0.049] |
| Perceived supervisor support | 0.013 | 0.008 | -0.021 |
|  | (0.038) | (0.023) | (0.060) |
|  | [0.726] | [0.729] | [0.726] |
| Patrol | 0.008 | 0.005 | -0.012 |
|  | (0.073) | (0.044) | (0.118) |
|  | [0.917] | [0.917] | [0.917] |
| Age | -0.001 | -0.001 | 0.002 |
|  | (0.004) | (0.002) | (0.006) |
|  | [0.781] | [0.776] | [0.779] |
| Female | -0.111 | -0.067 | 0.178 |
|  | (0.112) | (0.070) | (0.175) |
|  | [0.320] | [0.337] | [0.308] |
| Black | -0.045 | -0.028 | 0.073 |
|  | (0.091) | (0.075) | (0.164) |
|  | [0.622] | [0.706] | [0.657] |
| Hispanic | -0.053 | -0.036 | 0.089 |
|  | (0.087) | (0.081) | (0.165) |
|  | [0.544] | [0.654] | [0.591] |
| Other | -0.054 | -0.037 | 0.091 |
|  | (0.082) | (0.070) | (0.150) |
|  | [0.507] | [0.595] | [0.542] |
| Bachelor's degree or higher | -0.099 | -0.060 | 0.158 |
|  | (0.086) | (0.051) | (0.129) |
|  | [0.248] | [0.243] | [0.220] |
| Observations | 75 | 75 | 75 |
| Average marginal effects (AMEs) presented, robust standard errors, p-value in bracket; *Recoded: D = Disagree, N = Neutral, A = Agree. | | | |

| Table A10. 3-point Ordered Logistic Regression: Appropriateness | | | |
| --- | --- | --- | --- |
|  | y=D | y=N | y=A |
|  | b/se/p | b/se/p | b/se/p |
| CGPD (ref = AJPD) | -0.145 | -0.111 | 0.256 |
|  | (0.070) | (0.045) | (0.093) |
|  | [0.039] | [0.014] | [0.006] |
| Treatment assignment | -0.081 | -0.062 | 0.143 |
|  | (0.058) | (0.048) | (0.099) |
|  | [0.162] | [0.196] | [0.148] |
| Organizational justice | -0.070 | -0.053 | 0.123 |
|  | (0.045) | (0.038) | (0.076) |
|  | [0.123] | [0.158] | [0.107] |
| Perceived supervisor support | -0.012 | -0.009 | 0.022 |
|  | (0.032) | (0.024) | (0.056) |
|  | [0.704] | [0.700] | [0.701] |
| Patrol | 0.077 | 0.059 | -0.136 |
|  | (0.067) | (0.050) | (0.111) |
|  | [0.246] | [0.241] | [0.220] |
| Age | 0.001 | 0.000 | -0.001 |
|  | (0.003) | (0.002) | (0.006) |
|  | [0.849] | [0.851] | [0.850] |
| Female | -0.102 | -0.078 | 0.180 |
|  | (0.093) | (0.073) | (0.160) |
|  | [0.274] | [0.288] | [0.259] |
| Black | -0.000 | -0.000 | 0.001 |
|  | (0.079) | (0.072) | (0.151) |
|  | [0.996] | [0.996] | [0.996] |
| Hispanic | 0.008 | 0.007 | -0.015 |
|  | (0.078) | (0.063) | (0.141) |
|  | [0.915] | [0.911] | [0.913] |
| Other | 0.066 | 0.034 | -0.099 |
|  | (0.066) | (0.033) | (0.095) |
|  | [0.321] | [0.307] | [0.293] |
| Bachelor's degree or higher | -0.098 | -0.074 | 0.172 |
|  | (0.073) | (0.057) | (0.121) |
|  | [0.182] | [0.191] | [0.157] |
| Observations | 76 | 76 | 76 |
| Average marginal effects (AMEs) presented, robust standard errors, p-value in bracket; *Recoded: D = Disagree, N = Neutral, A = Agree. | | | |

| Table A11. 3-point Ordered Logistic Regression: Feasibility | | | |
| --- | --- | --- | --- |
|  | y=D | y=N | y=A |
|  | b/se/p | b/se/p | b/se/p |
| CGPD (ref = AJPD) | -0.063 | -0.102 | 0.166 |
|  | (0.044) | (0.045) | (0.081) |
|  | [0.150] | [0.024] | [0.040] |
| Treatment assignment | -0.098 | -0.159 | 0.258 |
|  | (0.038) | (0.084) | (0.104) |
|  | [0.010] | [0.058] | [0.013] |
| Organizational justice | -0.061 | -0.098 | 0.159 |
|  | (0.033) | (0.051) | (0.073) |
|  | [0.063] | [0.055] | [0.030] |
| Perceived supervisor support | -0.015 | -0.024 | 0.039 |
|  | (0.022) | (0.032) | (0.054) |
|  | [0.502] | [0.458] | [0.468] |
| Patrol | 0.022 | 0.036 | -0.058 |
|  | (0.038) | (0.062) | (0.098) |
|  | [0.562] | [0.563] | [0.558] |
| Age | 0.002 | 0.003 | -0.004 |
|  | (0.002) | (0.004) | (0.006) |
|  | [0.461] | [0.472] | [0.459] |
| Female | -0.052 | -0.083 | 0.135 |
|  | (0.080) | (0.137) | (0.214) |
|  | [0.521] | [0.541] | [0.528] |
| Black | -0.045 | -0.118 | 0.163 |
|  | (0.041) | (0.152) | (0.188) |
|  | [0.275] | [0.436] | [0.386] |
| Hispanic | -0.047 | -0.131 | 0.178 |
|  | (0.033) | (0.104) | (0.130) |
|  | [0.157] | [0.207] | [0.171] |
| Other | 0.048 | 0.047 | -0.095 |
|  | (0.054) | (0.051) | (0.102) |
|  | [0.372] | [0.364] | [0.354] |
| Bachelor's degree or higher | -0.021 | -0.035 | 0.056 |
|  | (0.047) | (0.075) | (0.121) |
|  | [0.653] | [0.644] | [0.645] |
| Observations | 76 | 76 | 76 |
| Average marginal effects (AMEs) presented, robust standard errors, p-value in brackets; *Recoded: D = Disagree, N = Neutral, A = Agree. | | | |

Table A12. OLS Regression Models: Acceptability, Appropriateness, Feasibility

|  | (1) | (2) | (3) |
| --- | --- | --- | --- |
|  | Acceptability | Appropriateness | Feasibility |
|  | b/se/p | b/se/p | b/se/p |
| CGPD (ref = AJPD) | 0.451 | 0.532 | 0.342 |
|  | (0.213) | (0.214) | (0.169) |
|  | [0.038] | [0.015] | [0.047] |
|  |  |  |  |
| Treatment assignment | 0.176 | 0.185 | 0.293 |
|  | (0.223) | (0.201) | (0.182) |
|  | [0.433] | [0.362] | [0.113] |
|  |  |  |  |
| Organizational justice | 0.317 | 0.265 | 0.226 |
|  | (0.195) | (0.151) | (0.140) |
|  | [0.109] | [0.084] | [0.111] |
|  |  |  |  |
| Perceived supervisor support | 0.068 | 0.081 | 0.141 |
|  | (0.140) | (0.119) | (0.117) |
|  | [0.629] | [0.496] | [0.232] |
|  |  |  |  |
| Patrol | -0.082 | -0.241 | -0.133 |
|  | (0.243) | (0.208) | (0.188) |
|  | [0.737] | [0.251] | [0.480] |
|  |  |  |  |
| Age | 0.003 | -0.003 | -0.005 |
|  | (0.012) | (0.011) | (0.011) |
|  | [0.800] | [0.821] | [0.638] |
|  |  |  |  |
| Female | 0.331 | 0.373 | 0.233 |
|  | (0.316) | (0.284) | (0.298) |
|  | [0.300] | [0.194] | [0.438] |
|  |  |  |  |
| Black | 0.118 | -0.037 | 0.203 |
|  | (0.303) | (0.288) | (0.276) |
|  | [0.698] | [0.899] | [0.465] |
|  |  |  |  |
| Hispanic | 0.207 | -0.185 | 0.247 |
|  | (0.326) | (0.273) | (0.236) |
|  | [0.527] | [0.501] | [0.298] |
|  |  |  |  |
| Other | 0.042 | -0.322 | -0.106 |
|  | (0.349) | (0.271) | (0.201) |
|  | [0.904] | [0.239] | [0.601] |
|  |  |  |  |
| Bachelor's degree or higher | 0.274 | 0.247 | 0.156 |
|  | (0.294) | (0.221) | (0.261) |
|  | [0.354] | [0.268] | [0.553] |
|  |  |  |  |
| Constant | 2.734 | 3.128 | 3.206 |
|  | (0.633) | (0.535) | (0.543) |
|  | [0.000] | [0.000] | [0.000] |
| Observations | 75 | 76 | 76 |
| F | 1.717 | 2.070 | 3.685 |
| R2 | 0.212 | 0.248 | 0.307 |
| RMSE | 0.871 | 0.786 | 0.691 |
| *5-point Likert scale outcomes (1 = Strongly disagree, 3 = Neutral, 5 = Strongly agree); Robust standard errors in parentheses; p-values in brackets. | | | |
